# Chemotaxis when Bacteria Remember: Drift versus Diffusion

**DOI:** 10.1371/journal.pcbi.1002283

**Published:** 2011-12-01

**Authors:** Sakuntala Chatterjee, Rava Azeredo da Silveira, Yariv Kafri

**Affiliations:** 1 Department of Physics, Technion, Haifa, Israel; 2 Department of Physics and Department of Cognitive Studies, École Normale Supérieure, Paris, France; 3 Laboratoire de Physique Statistique, Centre National de la Recherche Scientifique, Université Pierre et Marie Curie, Université Denis Diderot, Paris, France; University of Illinois at Urbana-Champaign, United States of America

## Abstract

*Escherichia coli* (*E. coli*) bacteria govern their trajectories by switching between running and tumbling modes as a function of the nutrient concentration they experienced in the past. At short time one observes a drift of the bacterial population, while at long time one observes accumulation in high-nutrient regions. Recent work has viewed chemotaxis as a compromise between drift toward favorable regions and accumulation in favorable regions. A number of earlier studies assume that a bacterium resets its memory at tumbles – a fact not borne out by experiment – and make use of approximate coarse-grained descriptions. Here, we revisit the problem of chemotaxis without resorting to any memory resets. We find that when bacteria respond to the environment in a non-adaptive manner, chemotaxis is generally dominated by diffusion, whereas when bacteria respond in an adaptive manner, chemotaxis is dominated by a bias in the motion. In the adaptive case, favorable drift occurs together with favorable accumulation. We derive our results from detailed simulations and a variety of analytical arguments. In particular, we introduce a new coarse-grained description of chemotaxis as biased diffusion, and we discuss the way it departs from older coarse-grained descriptions.

## Author Summary

The chemotaxis of *Escherichia coli* is a prototypical model of navigational strategy. The bacterium maneuvers by switching between near-straight motion, termed runs, and tumbles which reorient its direction. To reach regions of high nutrient concentration, the run-durations are modulated according to the nutrient concentration experienced in recent past. This navigational strategy is quite general, in that the mathematical description of these modulations also accounts for the active motility of *C. elegans* and for thermotaxis in *Escherichia coli*. Recent studies have pointed to a possible incompatibility between reaching regions of high nutrient concentration quickly and staying there at long times. We use numerical investigations and analytical arguments to reexamine navigational strategy in bacteria. We show that, by accounting properly for the full memory of the bacterium, this paradox is resolved. Our work clarifies the mechanism that underlies chemotaxis and indicates that chemotactic navigation in wild-type bacteria is controlled by drift while in some mutant bacteria it is controlled by a modulation of the diffusion. We also propose a new set of effective, large-scale equations which describe bacterial chemotactic navigation. Our description is significantly different from previous ones, as it results from a conceptually different coarse-graining procedure.

## Introduction

The bacterium *E. coli* moves by switching between two types of motions, termed ‘run’ and ‘tumble’ [1]. Each results from a distinct movement of the flagella. During a run, flagella motors rotate counter-clockwise (when looking at the bacteria from the back), inducing an almost constant forward velocity of about 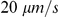, along a near-straight line. In an environment with uniform nutrient concentration, run durations are distributed exponentially with a mean value of about 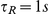 [2]. When motors turn clockwise, the bacterium undergoes a tumble, during which, to a good approximation, it does not translate but instead changes its direction randomly. In a uniform nutrient-concentration profile, the tumble duration is also distributed exponentially but with a much shorter mean value of about 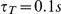 [3].

**Figure 1:**
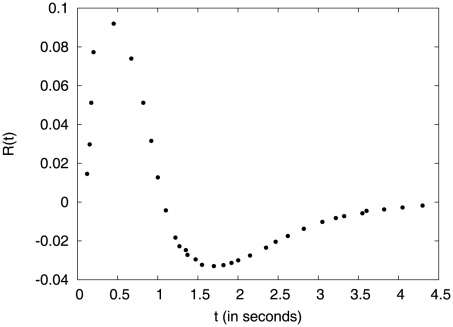
Bilobe response function of wild-type *E. coli* used in the numerics in **Fig. 3**. For the sake of computational simplicity, we have used a discrete sampling of the experimental data presented in Ref. [18] instead of working with the complete data set. This did not affect our conclusions.

When the nutrient (or, more generally, chemoattractant) concentration varies in space, bacteria tend to accumulate in regions of high concentration (or, equivalently, the bacteria can also be repelled by chemorepellants and tend to accumulate in low chemical concentration) [4]. This is achieved through a modulation of the run durations. The biochemical pathway that controls flagella dynamics is well understood [1], [5]–[7] and the stochastic ‘algorithm’ which governs the behavior of a single motor is experimentally measured. The latter is routinely used as a model for the motion of a bacteria with many motors [1], [8]–[11]. This algorithm represents the motion of the bacterium as a non-Markovian random walker whose stochastic run durations are modulated via a memory kernel, shown in Fig. 1. Loosely speaking, the kernel compares the nutrient concentration experienced in the recent past with that experienced in the more distant past. If the difference is positive, the run duration is extended; if it is negative, the run duration is shortened.

In a complex medium bacterial navigation involves further complications; for example, interactions among the bacteria, and degradations or other dynamical variations in the chemical environment. These often give rise to interesting collective behavior such as pattern formation [12], [13]. However, in an attempt to understand collective behavior, it is imperative to first have at hand a clear picture of the behavior of a single bacterium in an inhomogeneous chemical environment. We are concerned with this narrower question in the present work.

Recent theoretical studies of single-bacterium behavior have shown that a simple connection between the stochastic algorithm of motion and the average chemotactic response is far from obvious [8]–[11]. In particular, it appeared that favorable chemotactic drift could not be reconciled with favorable accumulation at long times, and chemotaxis was viewed as resulting from a compromise between the two [11]. The optimal nature of this compromise in bacterial chemotaxis was examined in Ref. [10]. In various approximations, while the negative part of the response kernel was key to favorable accumulation in the steady state, it suppressed the drift velocity. Conversely, the positive part of the response kernel enhanced the drift velocity but reduced the magnitude of the chemotactic response in the steady state.

Here, we carry out a detailed study of the chemotactic behavior of a single bacterium in one dimension. We find that, for an ‘adaptive’ response kernel (*i.e.*, when the positive and negative parts of the response kernel have equal weight such that the total area under the curve vanishes), there is no incompatibility between a strong steady-state chemotaxis and a large drift velocity. A strong steady-state chemotaxis occurs when the positive peak of the response kernel occurs at a time much smaller than 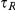 and the negative peak at a time much larger than 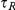, in line with experimental observation. Moreover, we obtain that the drift velocity is also large in this case. For a general ‘non-adaptive’ response kernel (*i.e.*, when the area under the response kernel curve is non-vanishing), however, we find that a large drift velocity indeed opposes chemotaxis. Our calculations show that, in this case, a position-dependent diffusivity is responsible for chemotactic accumulation.

In order to explain our numerical results, we propose a simple coarse-grained model which describes the bacterium as a biased random walker with a drift velocity and diffusivity, both of which are, in general, position-dependent. This simple model yields good agreement with results of detailed simulations. We emphasize that our model is distinct from existing coarse-grained descriptions of *E. coli* chemotaxis [13]–[16]. In these, coarse-graining was performed over left- and right-moving bacteria separately, after which the two resulting coarse-grained quantities were then added to obtain an equation for the total coarse-grained density. We point out why such approaches can fail and discuss the differences between earlier models and the present coarse-grained model.

## Models

Following earlier studies of chemotaxis [9], [17], we model the navigational behavior of a bacterium by a stochastic law of motion with Poissonian run durations. A switch from run to tumble occurs during the small time interval between 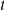 and 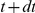 with a probability
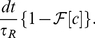
(1)Here, 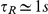 and 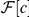 is a functional of the chemical concentration, 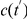, experienced by the bacterium at times 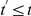. In shallow nutrient gradients, the functional can be written as
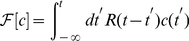
(2)The response kernel, 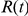, encodes the action of the biochemical machinery that processes input signals from the environment. Measurements of the change in the rotational bias of a flagellar motor in wild-type bacteria, in response to instantaneous chemoattractant pulses were reported in Refs. [17], [18]; experiments were carried out with a tethering assay. The response kernel obtained from these measurements has a bimodal shape, with a positive peak around 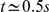 and a negative peak around 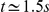 (see Fig. 1). The negative lobe is shallower than the positive one and extends up to 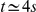, beyond which it vanishes. The total area under the response curve is close to zero. As in other studies of *E. coli* chemotaxis, we take this response kernel to describe the modulation of run duration of swimming bacteria [8]–[11]. Recent experiments suggest that tumble durations are not modulated by the chemical environment and that as long as tumbles last long enough to allow for the reorientation of the cell, bacteria can perform chemotaxis successfully [19], [20].

The model defined by Eqs. 1 and 2 is linear. Early experiments pointed to a non-linear, in effect a threshold-linear, behavior of a bacterium in response to chemotactic inputs [17], [18]. In these studies, a bacterium modulated its motion in response to a positive chemoattractant gradient, but not to a negative one. In the language of present model, such a threshold-linear response entails replacing the functional defined in Eq. 2 by zero whenever the integral is negative. More recent experiments suggest a different picture, in which a non-linear response is expected only for a strong input signal whereas the response to weak chemoattractant gradient is well described by a linear relation [21]. Here, we present an analysis of the linear model. For the sake of completeness, in Text S1, we present a discussion of models which include tumble modulations and a non-linear response kernel. Although recent experiments have ruled out the existence of both these effects in *E.coli* chemotaxis, in general such effects can be relevant to other systems with similar forms of the response function.

The shape of the response function hints to a simple mechanism for the bacterium to reach regions with high nutrient concentration. The bilobe kernel measures a temporal gradient of the nutrient concentration. According to Eq. 1, if the gradient is positive, runs are extended; if it is negative, runs are unmodulated. However, recent literature [8], [9], [11] has pointed out that the connection between this simple picture and a detailed quantitative analysis is tenuous. For example, de Gennes used Eqs. 1 to calculate the chemotactic drift velocity of bacteria [8]. He found that a singular kernel, 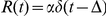, where 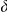 is a Dirac function and 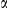 a positive constant, lead to a mean velocity in the direction of increasing nutrient concentration even when bacteria are memoryless (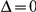). Moreover, any addition of a negative contribution to the response kernel, as seen in experiments (see Fig. 1), lowered the drift velocity. Other studies considered the steady-state density profile of bacteria in a container with closed walls, both in an approximation in which correlations between run durations and probability density were ignored [11] and in an approximation in which the memory of the bacterium was reset at run-to-tumble switches [9]. Both these studies found that, in the steady state, a negative contribution to the response function was mandatory for bacteria to accumulate in regions of high nutrient concentration. These results seem to imply that the joint requirement of favorable transient drift and steady-state accumulation is problematic. The paradox was further complicated by the observation [9] that the steady-state single-bacterium probability density was sensitive to the precise shape of the kernel: when the negative part of the kernel was located far beyond 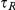 it had little influence on the steady-state distribution [11]. In fact, for kernels similar to the experimental one, model bacteria accumulated in regions with low nutrient concentration in the steady state [9].

## Results

### Simulations and analytical treatment of chemotactic bacterial accumulation

In order to resolve these paradoxes and to better understand the mechanism that leads to favorable accumulation of bacteria, we perform careful numerical studies of bacterial motion in one dimension. In conformity with experimental observations [17], [18], we do not make any assumption of memory reset at run-to-tumble switches.

We model a bacterium as a one-dimensional non-Markovian random walker. The walker can move either to the left or to the right with a fixed speed, 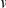, or it can tumble at a given position before initiating a new run. In the main paper, we present results only for the case of instantaneous tumbling with 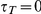, while results for non-vanishing 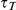 are discussed in Text S1. There, we verify that for an adaptive response kernel 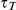 does not have any effect on the steady-state density profile. For a non-adaptive response kernel, the correction in the steady-state slope due to finite 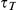 is small and proportional to 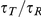.

The run durations are Poissonian and the tumble probability is given by Eq. 1. The probability to change the run direction after a tumble is assumed to have a fixed value, 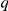, which we treat as a parameter. The specific choice of the value of 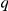 does not affect our broad conclusions. We find that, as long as 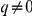, only certain detailed quantitative aspects of our numerical results depend on 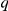. (See Text S1 for details on this point.) We assume that bacteria are in a box of size 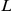 with reflecting walls and that they do not interact among each other. We focus on the steady-state behavior of a population. Reflecting boundary conditions are a simplification of the actual behavior [22], [23]; as long as the total ‘probability current’ (see discussion below) in the steady state vanishes, our results remain valid even if the walls are not reflecting.

As a way to probe chemotactic accumulation, we consider a linear concentration profile of nutrient: 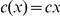. We work in a weak gradient limit, *i.e.*, the value of 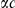 is chosen to be sufficiently small to allow for a linear response. Throughout, we use 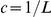 in our numerics. From the linearity of the problem, results for a different attractant gradient, 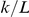, can be obtained from our results through a scaling factor 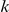. In the linear reigme, we obtain a spatially linear steady-state distribution of individual bacterium positions, or, equivalently, a linear density profile of a bacterial population. Its slope, which we denote by 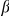, is a measure of the strength of chemotaxis. A large slope indicates strong bacterial preference for regions with higher nutrient concentration. Conversely, a vanishing slope implies that bacteria are insensitive to the gradient of nutrient concentration and are equally likely to be anywhere along the line. We would like to understand the way in which the slope 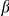 depends on the different time scales present in the system.

#### Results with non-adaptive response kernels

One particular advantage of a linear model is that a general problem can be solved by superposing the solutions of simpler problems–namely, with delta-function response kernels–with suitably chosen coefficients. Thus, solving the problem with a singular response kernel amounts to a full solution and we focus here on this case.

In our simulations, we start from an arbitrary bacterium position inside a box of size 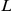. Each time step has a duration 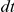, during which a running bacterium moves over a distance 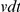. This distance corresponds to one lattice spacing in our model, in which a lattice is introduced because time is discretized. Throughout the numerics, we use 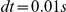 and 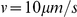, which means that the lattice spacing in our simulations is 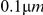. Results for different values of 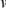 can be obtained by rescaling the lattice spacing accordingly. At the end of each time step, we compute the functional defined in Eq. 2; for a singular response kernel, 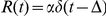, this takes the form 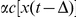, where 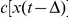 is the nutrient concentration experienced by the bacterium at time 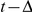. At the end of each time step the bacterium either tumbles, with a probability 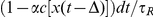, or continues to move in the same direction. At every tumble, the velocity of the bacterium is reversed with a probability 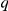.

The system reaches a steady state over a time scale which is of order 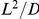, where the diffusivity is given by 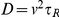. We verify numerically that after this time the bacterial density profile inside the box does not change further and assumes a time-independent linear form. We focus on the slope, 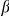, of this profile. For an experimental realization of the steady-state behavior of a single bacterium, we provide here an estimate of the time scales and length scales involved. Since the long-time behavior of the system is diffusive (see the discussion of the coarse-grained model below), the relaxation time is 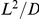. Our results on the steady-state distribution of bacteria hold, realistically, if this relaxation time does not exceed the typical division time of an *E. coli* bacterium, which is of the order of 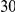 minutes. Substituting experimental values for the parameters, we find the description should be valid for system sizes 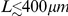. In our simulations, we use a somewhat larger system (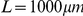) so as to have cleaner results with negligible effects of the reflecting walls at the two boundaries. (Numerics data show that the width of the boundary layer is about 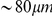.)

**Figure 2:**
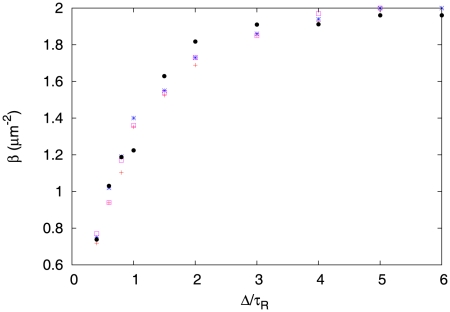
The slope 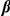
(scaled by a factor of 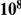) as a function of 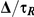
>, for the choice of response kernel 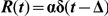. Note that for 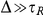 the slope saturates to a non-vanishing value. The symbols 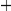, 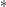, and □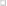 correspond to slopes measured in simulations with 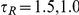, and 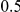 seconds, respectively. The black solid circles are derived from our coarse-grained formulation (Eq. 9). Here 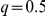, 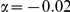, 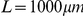, 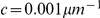, 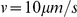.

According to our numerical simulations, for 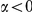, 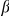 *increases* with 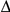 and displays a plateau for 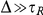 (Fig. 2). Simulations probing various values of 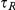 also confirmed that 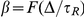, *i.e.*, that the slope is a scaling function of 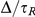. Clearly, for positive 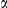 the sign of 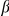 is simply reversed, which corresponds to an unfavorable chemotaxis [11], [14].

For small 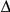, one can write down an approximate master equation for left-mover and right-mover densities and use it to show that the slope increases linearly with 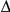 (see Text S1 for details). It is surprising, however, that the slope appears to saturate to a non-vanishing value for 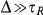. Indeed one would expect that, if the response kernel relies on a time much earlier than 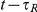, a large enough number of tumbles occur between this past time and the present time so as to eliminate any correlation between the nutrient concentration in the past and the present direction of motion. If this argument holds, one would expect that the slope 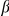 vanish for 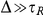. Below, we return to this argument and explain why it is misleading.

#### Results with adaptive response kernels

For wild-type bacteria, the total area under the response kernel vanishes (Fig. 1). As a result, their behavior is adaptive: chemotaxis is insensitive to the overall level of nutrient, but sensitive to spatial variations [17], [18]. In this section, before examining the case of a bilobe response kernel similar to the experimental one, we consider a toy model defined by the difference of two singular forms: 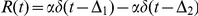, with 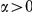. Because our problem is linear, the steady-state slope of bacterial density, 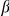, can be calculated from a simple linear superposition, as:
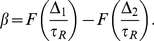
(3)Since the function 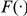 is monotonic, the absolute value of 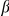 increases with the difference of 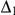 and 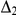. Strong chemotaxis occurs when 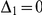 and 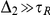.

**Figure 3:**
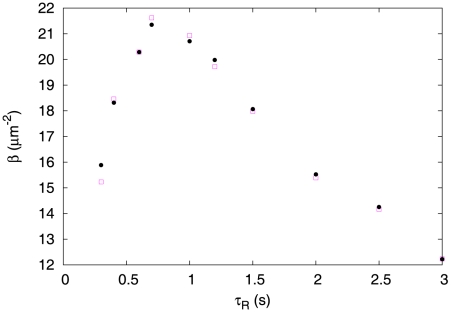
The slope 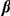 (scaled by a factor of 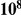) as a function of 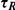
> for the experimental response kernel shown in Fig. 1. Open squares: numerical results from simulations. Solid circles: prediction of the coarse-grained model. Here, 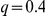, 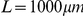, 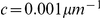, 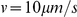.

We now turn to the experimental case of a bilobe response kernel. It is not computationally feasible to work with the complete set of experimental data [18], so we have used a discrete subset (Fig. 1) which we represent as a series of delta-functions. Given this approximate response kernel, we investigate the behavior of the slope as a function of 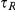. Based on our results for the case of two delta functions, we expect that chemotaxis be weak if 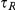 is either much smaller than the delay of the positive peak in the response kernel or much larger than the delay of the negative peak. We expect optimum chemotaxis for a value of 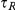 that falls in between the two delays. We verify this prediction in Fig. 3 (in the linear model). We note that the maximum slope occurs for a value of 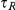 close to the experimentally recorded value of about 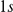.

### Coarse-grained description of chemotaxis as diffusion with drift

In order to gain insight into our numerical results, we developed a simple coarse-grained model of chemotaxis. For the sake of simplicity, we first present the model for a non-adaptive, singular response kernel, 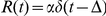, and, subsequently, we generalize the model to adaptive response kernels by making use of linear superposition.

**Figure 4:**
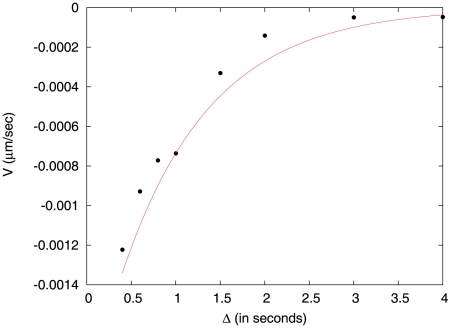
The chemotactic drift velocity, 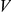, as a function of 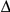, for the response kernel. 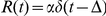. Solid circles: numerical results. Line: approximate analytical results from [8]. 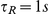 and other numerical parameters as in Fig. 2.

The memory trace embodied by the response kernel induces temporal correlations in the trajectory of the bacterium. However, if we consider the coarse-grained motion of the bacterium over a spatial scale that exceeds the typical run stretch and a temporal scale that exceeds the typical run duration, then we can assume that it behaves as a Markovian random walker with drift velocity 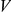 and diffusivity 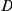. Since the steady-state probability distribution, 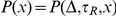, is flat for 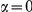, for small 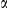 we can write
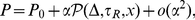
(4)

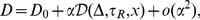
(5)

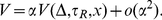
(6)Here, 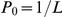 and 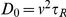. Since we are neglecting all higher order corrections in 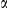, our analysis is valid only when 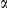 is sufficiently small. In particular, even when 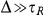, we assume that the inequality 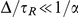 is still satisfied. The chemotactic drift velocity, 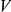, vanishes if 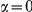; it is defined as the mean displacement per unit time of a bacterium starting a new run at a given location. Clearly, even in the steady state when the current 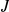, defined through 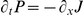, vanishes, 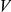 may be non-vanishing (see Eq. 8 below). In general, the non-Markovian dynamics make 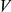 dependent on the initial conditions. However, in the steady state this dependence is lost and 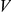 can be calculated, for example, by performing a weighted average over the probability of histories of a bacterium. This is the quantity that is of interest to us. An earlier calculation by de Gennes showed that, if the memory preceding the last tumble is ignored, then for a linear profile of nutrient concentration the drift velocity is independent of position and takes the form 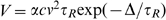 [8]. While the calculation applies strictly in a regime with 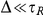 (because of memory erasure), in fact its result captures the behavior well over a wide range of parameters (see Fig. 4). To measure 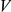 in our simulations, we compute the average displacement of the bacterium between two successive tumbles in the steady state, and we extract therefrom the drift velocity. (For details of the derivation, see Text S1.) We find that 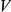 is negative for 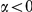 and that its magnitude falls off with increasing values of 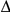 (Fig. 4). We also verify that 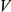 indeed does not show any spatial dependence (data shown in Fig. 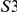 of Text S1). We recall that, in our numerical analysis, we have used a small value of 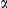; this results in a low value of 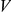. We show below that for an experimentally measured bilobe response kernel, obtained by superposition of singular response kernels, the magnitude of 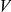 becomes larger and comparable with experimental values.

**Figure 5:**
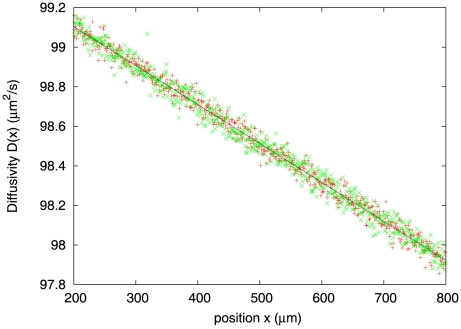
The diffusivity, 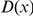, as a function of position, 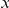, for the response kernel 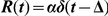 with 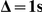 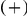 and 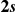 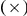. Instead of plotting 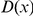 for the entire range of 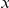, we leave out boundary regions to avoid the effect of the reflecting walls. (From the numerics, the width of the boundary layer is 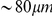.) 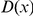 falls off linearly with 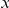 and is independent of 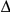. Data fitting yields 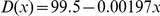 and the coarse-grained model predicts 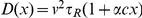. For the chosen set of parameters, 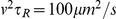 and the 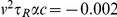. The discrepancy between the numerical and the predicted slopes is due to higher-order corrections in 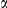, while discretization of space in simulations causes the slight mismatch in the constant term. 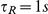 and other numerical parameters are as in Fig. 2.

To obtain the diffusivity, 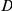, we first calculate the effective mean free path in the coarse-grained model. The tumbling frequency of a bacterium is 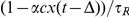 and depends on the details of its past trajectory. In the coarse-grained model, we replace the quantity 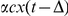 by an average 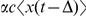 over all the trajectories within the spatial resolution of the coarse-graining. Equivalently, in a population of non-interacting bacteria, the average is taken over all the bacteria contained inside a blob, and, hence, 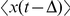 denotes the position of the center of mass of the blob at a time 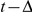 in the past. As mentioned above, the drift velocity is proportional to 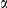, so that 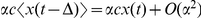. The average tumbling frequency then becomes 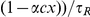 and, consequently, the mean free path becomes 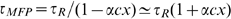. As a result, the diffusivity is expressed as 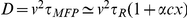. We checked this form against our numerical results (Fig. 5).

Having evaluated the drift velocity, 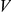, and the diffusivity, 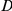, we now proceed to write down the continuity equation (for a more rigorous but less intuitive approach, see [10]). For a biased random walker on a lattice, with position-dependent hopping rates 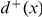 and 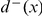 towards the right and the left, respectively, one has 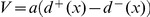 and 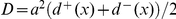, where 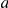 is the lattice constant. In the continuum limit, the temporal evolution of the probability density is given by a probability current, as
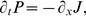
(7)where the current takes the form
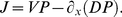
(8)For reflecting boundary condition, 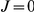 in the steady state. This constraint yields a steady-state slope
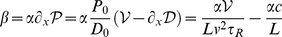
(9)for small 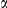. We use our measured values for 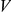 and 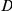 (Figs. 4 and 5), and compute the slope using Eq. 9. (For details of the measurement of 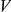, see Text S1.) We compare our analytical and numerical results in Fig. 2, which exhibits close agreement.

According to Eq. 9, steady-state chemotaxis results from a competition between drift motion and diffusion. For 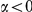, the drift motion is directed toward regions with a lower nutrient concentration and hence opposes chemotaxis. Diffusion is spatially dependent and becomes small for large nutrient concentrations (again for 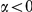), thus increasing the effective residence time of the bacteria in favorable regions. For large values of 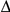, the drift velocity vanishes and one has a strong chemotaxis as 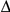 increases (Fig. 2). Finally, for 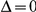, the calculation by de Gennes yields 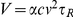 which exactly cancels the spatial gradient of 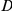 (to linear order in 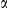), and there is no accumulation [8], [11].

These conclusions are easily generalized to adaptive response functions. For 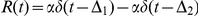, within the linear response regime, the effective drift velocity and diffusivity can be constructed by simple linear superposition: The drift velocity reads 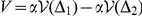. Interestingly, the spatial dependence of 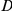 cancels out and 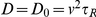. The resulting slope then depends on the drift only and is calculated as
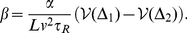
(10)In this case, the coarse-grained model is a simple biased random walker with constant diffusivity. For 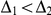 and 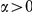, the net velocity, proportional to 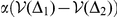, is positive and gives rise to a favorable chemotactic response, according to which bacteria accumulate in regions with high food concentration. Moreover, the slope increases as the separation between 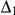 and 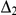 grows. We emphasize that there is no incompatibility between strong steady-state chemotaxis and large drift velocity. In fact, in the case of an adaptive response function, strong chemotaxis occurs only when the drift velocity is large.

For a bilobe response kernel, approximated by a superposition of many delta functions (Fig. 1), the slope, 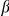, can be calculated similarly and in Fig. 3 we compare our calculation to the simulation results. We find close agreement in the case of a linear model with a bilobe response kernel and, in fact, also in the case of a non-linear model (see Text S1).

The experimental bilobe response kernel 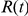 is a smooth function, rather than a finite sum of singular kernels over a set of discrete 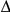 values (as in Fig. 1). Formally, we integrate singular kernels over a continuous range of 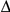 to obtain a smooth response kernel. If we then integrate the expression for the drift velocity obtained by de Gennes, according to this procedure, we find an overall drift velocity 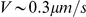, for the concentration gradient considered (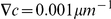). By scaling up the concentration gradient by a factor of 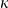, the value of 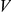 can also be scaled up by 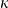 and can easily account for the experimentally measured velocity range.

## Discussion

We carried out a detailed analysis of steady-state bacterial chemotaxis in one dimension. The chemotactic performance in the case of a linear concentration profile of the chemoattractant, 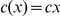, was measured as the slope of the bacterium probability density profile in the steady state. For a singular impulse response kernel, 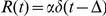, the slope was a scaling function of 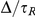, which vanished at the origin, increased monotonically, and saturated at large argument. To understand these results we proposed a simple coarse-grained model in which bacterial motion was described as a biased random walk with drift velocity, 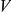, and diffusivity, 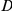. We found that for small enough values of 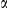, 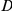 was independent of 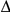 and varied linearly with nutrient concentration. By contrast, 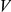 was spatially uniform and its value decreased monotonically with 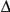 and vanished for 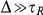. We presented a simple formula for the steady-state slope in terms of 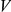 and 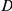. The prediction of our coarse-grained model agreed closely with our numerical results. Our description is valid when 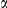 is small enough, and all our results are derived to linear order in 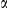. We assume 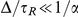 is always satisfied.

Our results for an impulse response kernel can be easily generalized to the case of response kernels with arbitrary shapes in the linear model. For an adaptive response kernel, the spatial dependence of the diffusivity, 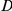, cancels out but a positive drift velocity, 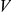, ensures bacterial accumulation in regions with high nutrient concentration, in the steady state. In this case, the slope is directly proportional to the drift velocity. As the delay between the positive and negative peaks of the response kernel grows, the velocity increases, with consequent stronger chemotaxis.

Earlier studies of chemotaxis [13]–[16] put forth a coarse-grained model different from ours. In the model first proposed by Schnitzer for a single chemotactic bacterium [14], he argued that, in order to obtain favorable bacterial accumulation, tumbling rate and ballistic speed of a bacterium must both depend on the direction of its motion. In his case, the continuity equation reads
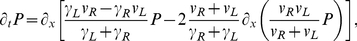
(11)where 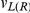 is the ballistic speed and 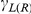 is the tumbling frequency of a bacterium moving toward the left (right). For *E. coli*, as discussed above, 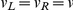, a constant independent of the location. In that case, Eq. 11 predicts that in order to have a chemotactic response in the steady state, one must have a non-vanishing drift velocity, *i.e.*, 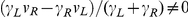. This contradicts our findings for non-adaptive response kernels, according to which a drift velocity only hinders the chemotactic response. The spatial variation of the diffusivity, instead, causes the chemotactic accumulation. This is not captured by Eq. 11. In the case of adaptive response kernels, the diffusivity becomes uniform while the drift velocity is positive, favoring chemotaxis. Comparing the expression of the flux, 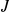, obtained from Eqs. 7 and 8 with that from Eq. 11, and matching the respective coefficients of 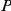 and 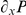, we find 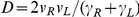 and 
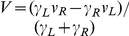
. As we argued above in discussing the coarse-grained model for adaptive response kernels, both 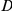 and 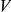 are spatially independent. This puts strict restrictions on the spatial dependence of 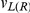 and 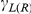. For example, as in *E. coli* chemotaxis 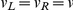, our coarse-grained description is recovered only if 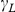 and 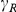 are also independent of 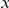.

We comment on a possible origin of the discrepancy between our work and earlier treatments. In Ref. [14], a continuity equation was derived for the coarse-grained probability density of a bacterium, starting from a pair of approximate master equations for the probability density of a right-mover and a left-mover, respectively. As the original process is non-Markovian, one can expect a master equation approach to be valid only at scales that exceed the scale over which spatiotemporal correlations in the behavior of the bacterium are significant. In particular, a biased diffusion model can be viewed as legitimate only if the (coarse-grained) temporal resolution allows for multiple runs and tumbles. If so, at the resolution of the coarse-grained model, left- and right-movers become entangled, and it is not possible to perform a coarse-graining procedure on the two species separately. Thus one cannot define probability densities for a left- and a right-mover that evolves in a Markovian fashion. In our case, left- and right-movers are coarse-grained simultaneously, and the total probability density is Markovian. Thus, our diffusion model differs from that of Ref. [14] because it results from a different coarse-graining procedure. The model proposed in Ref. [14] has been used extensively to investigate collective behaviors of *E. coli* bacteria such as pattern formation [13], [15], [16]. It would be worth asking whether the new coarse-grained description can shed new light on bacterial collective behavior.

## Supporting Information

Text S1 Chemotaxis when Bacteria Remember: Drift versus Diffusion (Supporting Information).
(PDF)
