# Supplementary material for: Chemotaxis when Bacteria Remember: Drift versus Diffusion
Source: PLoS Comput Biol. 2011 Dec 1;7(12):e1002283. doi: 10.1371/journal.pcbi.1002283 (PMC3228785; doi:10.1371/journal.pcbi.1002283)
Supplement: Text S1 — Chemotaxis when Bacteria Remember: Drift versus Diffusion (Supporting Information). (PDF) [file pcbi.1002283.s001.pdf]

Chemotaxis When Bacteria Remember: Drift versus Diffusion (Supporting Information)

Sakuntala Chatterjee(1), Rava Azeredo da Silveira(2,3) and Yariv Kafri(1)

(1) Department of Physics, Technion, Haifa-32000, Israel

(2) Department of Physics and Department of Cognitive Studies,

École Normale Supérieure, 24, rue Lhomond, 75005 Paris, France

(3) Laboratoire de Physique Statistique,

Centre National de la Recherche Scientifique,

Université Pierre et Marie Curie, Université Denis Diderot, France

I. SMALL-ARGUMENT BEHAVIOR OF $F(\Delta/\tau_R)$

Here, we argue that for small Δ/τ_R the function $F(\Delta/\tau_R)$ is linear. First, note that for an impulse response kernel, $R(t) = \alpha\delta(t-\Delta)$ the tumbling probability during the interval $[t, t+dt]$ is $[1 - \alpha c x(t-\Delta)]\, dt/\tau_R$. For small Δ, we can assume that no tumbling occurs during the interval $[t-\Delta, t]$. Then the effective tumbling rates become $[1 - \alpha c(x - v\Delta)]/\tau_R$ for right-movers and $[1 - \alpha c(x + v\Delta)]/\tau_R$ for left-movers. Based on this observation, we can write a pair of master equations that govern the densities of left-movers and right-movers, and we can solve them in the steady state. We obtain the slope, β, expressed as

$$\beta = -\frac{2\alpha c\Delta}{L\tau_R}. \tag{1}$$

Thus, in the limit $\Delta \ll \tau_R$ the scaling function $F(\Delta/\tau_R)$ depends linearly on its argument.

II. CHEMOTACTIC DRIFT VELOCITY AS A FUNCTION OF POSITION

Here, we show a sample of our numerical results on the drift velocity V as a function of position. We measure the average displacement of a bacterium in the steady state (see the corresponding discussion in the paper), and we compute V therefrom. Specifically, we perform the following procedure. We denote by $m_L(x)$ and $m_R(x)$ the total number of leftward and rightward runs, respectively, that initiate at the position x, at a time t, for a population of non-interacting bacteria. These quantities are well-defined in the context of

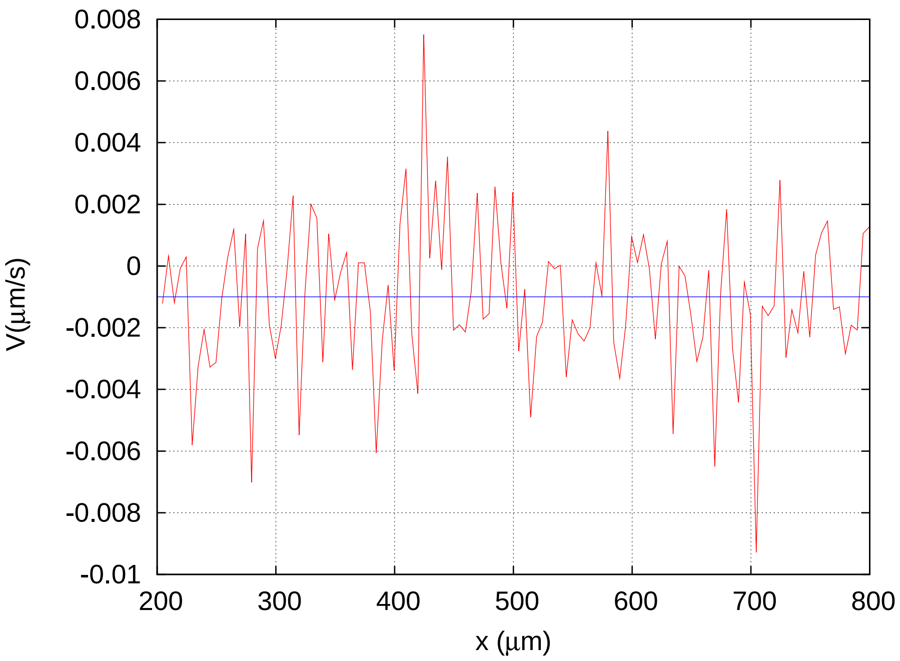

FIG. 1: *Drift velocity, V, as a function of position, x, for the case of a singular response kernel $R(t) = \alpha\delta(t - \Delta)$ (red line). Instead of showing the plot for the entire range of x, we leave out boundary regions to discard the effect of the reflecting walls. Our numerics show that the width of the boundary layer is $\sim 80\mu m$. Here, $L = 1000\mu m$, $q = 1.0$, $\tau_R = 1s$, $\Delta = 1s$, $\tau_T = 0$, $v = 10\mu m/s$, $\alpha = -0.1$, $c = 0.001\mu m^{-1}$. Based on the data shown, the drift velocity is $0.001 \pm 0.0001\mu m/s$ (blue line).*

our numerics because space is discretized. Furthermore, let $S_L(x)$ and $S_R(x)$ be the total leftward and rightward displacement, respectively, of the bacteria that undergo these runs. The average displacement per run is then given by $[S_R(x) - S_L(x)]/[m_R(x) + m_L(x)]$. Of course, the choice of the time scale as the duration of a run is arbitrary and other choices are equally valid. To leading order, this average displacement is linear in α. The drift velocity V, to order α, is then obtained by dividing this average displacement by τ_R. (Note that any $O(\alpha)$ correction to τ_R leads to $O(\alpha^2)$ correction to V, which we ignore here.) We find that up to the noise present in our numerical measurement, V does not display any dependence on position. An example is illustrated in Fig. 1.

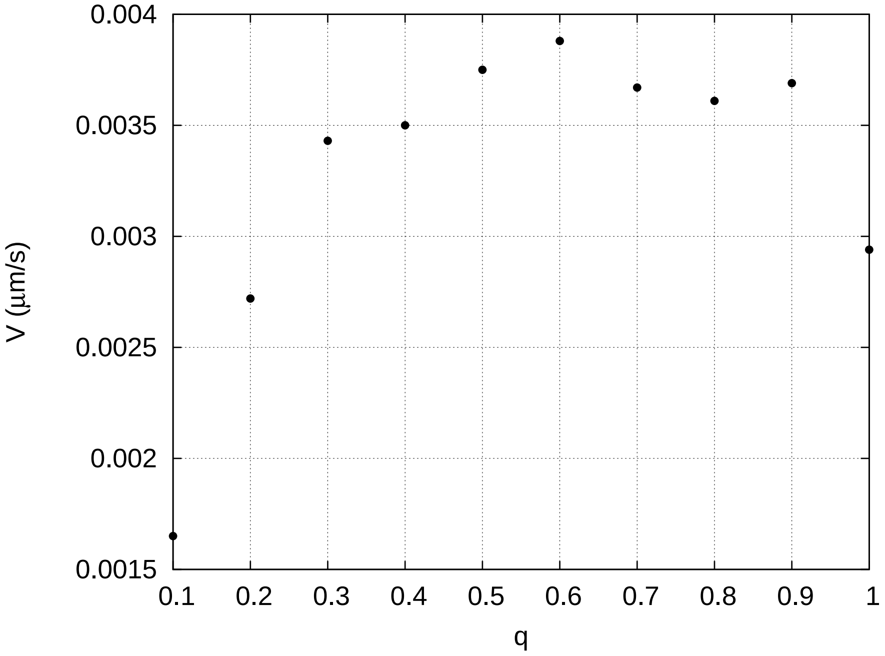

FIG. 2: *The drift velocity, V, as a function of the turning probability, q, for the case of a singular adaptive response kernel $R(t) = \alpha\delta(t - \Delta_1) - \alpha\delta(t - \Delta_2)$. Here, $L = 1000\mu m$, $\tau_R = 1s$, $\Delta_1 = 0.5s$, $\Delta_2 = 1.5s$, $\tau_T = 0$, $v = 10\mu m/s$, $\alpha = 0.1$, $c = 0.001\mu m^{-1}$.*

III. DEPENDENCE OF V ON THE TURNING PROBABILITY q

In our model, q denotes the turning probability, *i.e.*, the probability that the run direction inverses after a tumble. Our numerical explorations indicate that changing the value of q does not affect the qualitative behavior of the system. However, the numerical value of the drift velocity, and the value of β depend on q. In Fig. 2, we exhibit the variation of V as a function of q, in the case of an adaptive response function.

IV. RESULTS IN THE NON-LINEAR MODEL

Some earlier experiments indicate that bacteria modulate their run durations in response to a positive concentration gradient, but not to a negative one. In order to incorporate this feature in our model, we have to go beyond the linear response regime. In the non-linear model, whenever the linear functional (Eq. 2) becomes negative, it is replaced by 0. This is the only difference with the linear model. Thus, for a purely positive response kernel the

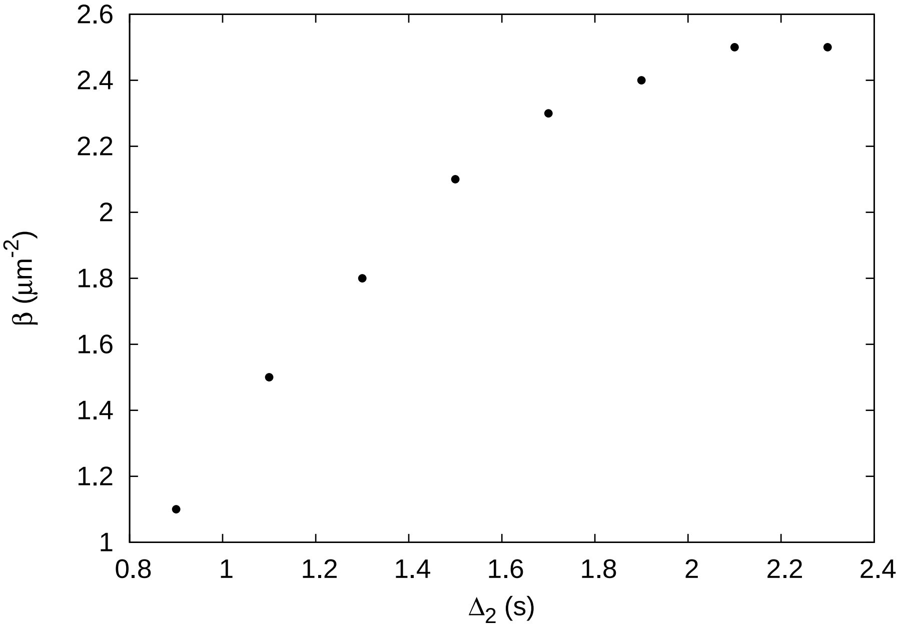

FIG. 3: The slope, β (scaled by a factor of 10^8), as a function of Δ_2, for $\Delta_1 = 0.5s$, in a non-linear model with balanced response kernel, $R(t) = \alpha\delta(t-\Delta_1) - \alpha\delta(t-\Delta_2)$. As in the linear model, β increases with the difference of Δ_1 and Δ_2. Here, $\tau_R = 1$ sec, $q = 0.4$, $\alpha = 0.1$, $L = 1000\mu m$, $c = 0.001\mu m^{-1}$, $v = 10\mu m/s$.

non-linear model behaves identically to the linear model, while for a purely negative response kernel the non-linear model displays no chemotaxis whatsoever. Hereafter, we examine only adaptive response kernels with balanced positive and negative contributions.

We first consider the idealized response kernel made of the superposition of positive and negative delta functions, $R(t) = \alpha\delta(t-\Delta_1) - \alpha\delta(t-\Delta_2)$. Simulations show that many qualitative features of the linear model still hold in the non-linear model. The scaling form valid in the linear case breaks down in the non-linear case, but the slope β increases with the separation between Δ_1 and Δ_2 and ultimately saturates to a non-vanishing value. Figures 3 and 4 display results of simulations. As expected, strong chemotaxis occurs when $\Delta_1 = 0$ and Δ_2 is substantially larger than τ_R. We have also verified that tumbling does not have much of an effect on the slope.

In the experimental case of a bilobe response kernel (Fig. 1 in the paper), we find that strong chemotaxis occurs when τ_R lies between the positive and the negative peaks of the response kernel, as found in the linear case. For smaller or larger values of τ_R,

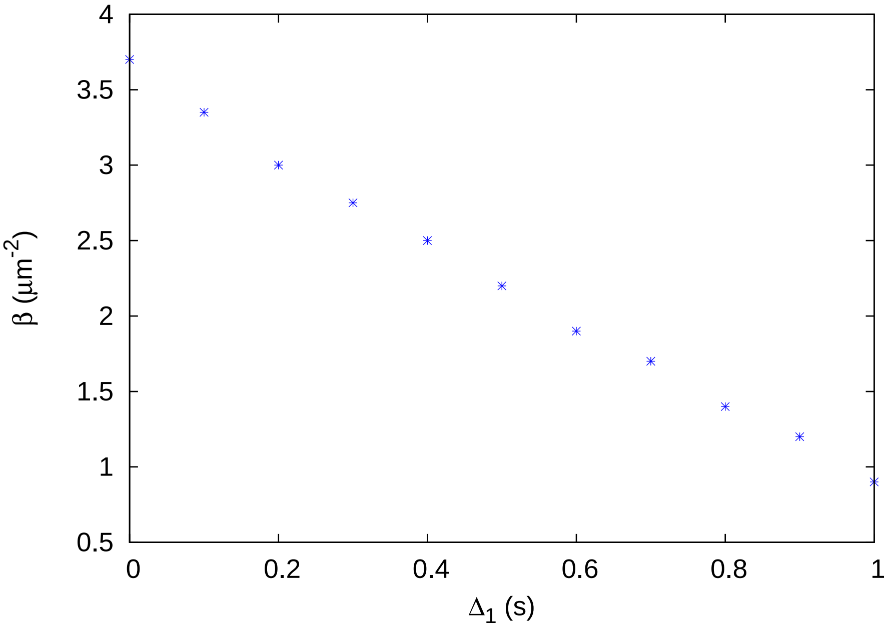

FIG. 4: The slope, β (scaled by a factor of 10^8), as a function of Δ_1, for $\Delta_2 = 1.5s$, in a non-linear model with balanced response kernel, $R(t) = \alpha\delta(t - \Delta_1) - \alpha\delta(t - \Delta_2)$. Numerical parameters are as in Fig. 3.

chemotaxis becomes weak. Figures 5 and 6 show our numerical results for $q = 0.4$ and $q = 0.5$, respectively. We note that, in both plots the value of τ_R for which the slope is maximum falls close to the experimental value of about $1s$. However, the exact position of the maximum depends on q. For $q = 0.4$ (Fig.5) maximum occurs at $\tau_R \simeq 0.8s$, while for $q = 0.5$ (Fig. 6) the maximum occurs at $\tau_R \simeq 1s$.

V. CHEMOTAXIS WITH NON-VANISHING TUMBLING DURATIONS

During a tumbling event the bacterium rotates about itself in a random fashion without any significant displacement. In a homogeneous nutrient concentration the average tumbling duration is $0.1s$, which is much smaller than the average run duration of $1s$. In the steady state one therefore expects that the bacterium spends only a fraction $\tau_T/\tau_R \simeq 0.1$ of the time in the tumbling state. For this reason, studies of chemotaxis often assume instantaneous tumbling.

It was shown recently that the existence of non-vanishing tumbling duration can yield

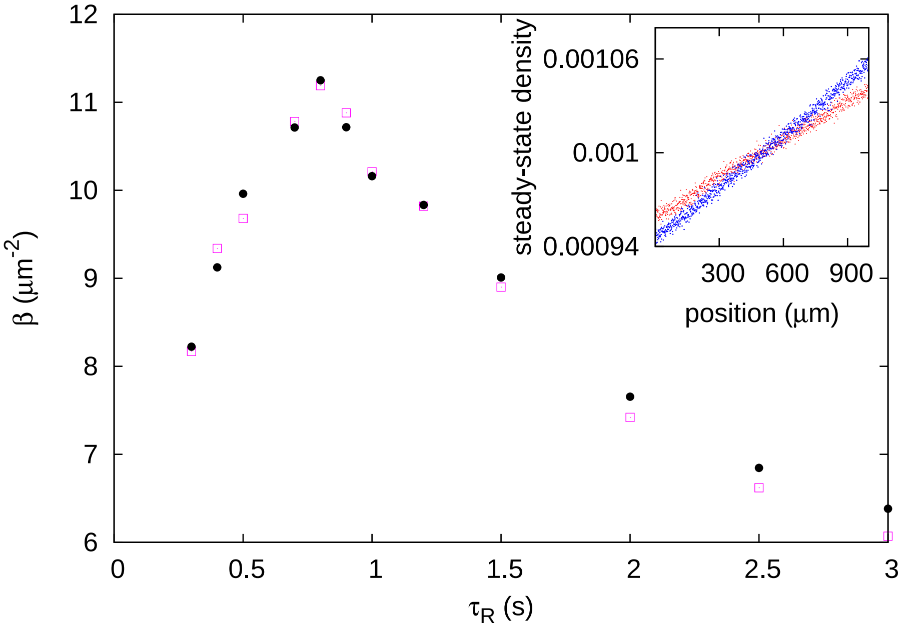

FIG. 5: The slope, β (scaled by a factor of 10^8), as a function of τ_R, in a non-linear model with the experimental bilobe response kernel of Fig. 1 in the paper. Open squares: numerical results from simulations. Solid circles: prediction of the coarse-grained model. Numerical parameters as in Fig. 3 in the paper. The inset shows the steady-state density profiles of the bacterial population for $\tau_R = 0.3, 0.8s$ (red and blue curve), respectively.

interesting results: even with a punctual response kernel, $R(t) = \alpha\delta(t-\Delta)$ with $\Delta = 0$, *i.e.* a memoryless bacterium, one can observe a chemotactic response (Kafri *et al.*, 2008). Here, we provide a simplified derivation of the steady-state density profile in this Markovian limit. The result will prove useful also for the analysis of the non-Markovian case with $\Delta \neq 0$.

Let $L(x,t)$ and $R(x,t)$ be the density of left-movers and right-movers, respectively, at location x and time t. We denote by $T^R(x,t)$ and $T^L(x,t)$ the densities of tumblers that were moving to the right and left, respectively, before tumbling. For $\Delta = 0$, the time evolution of these quantities can be described by master equations. In the case in which tumble durations are not modulated and tumble-to-run switches always occur at a fixed rate

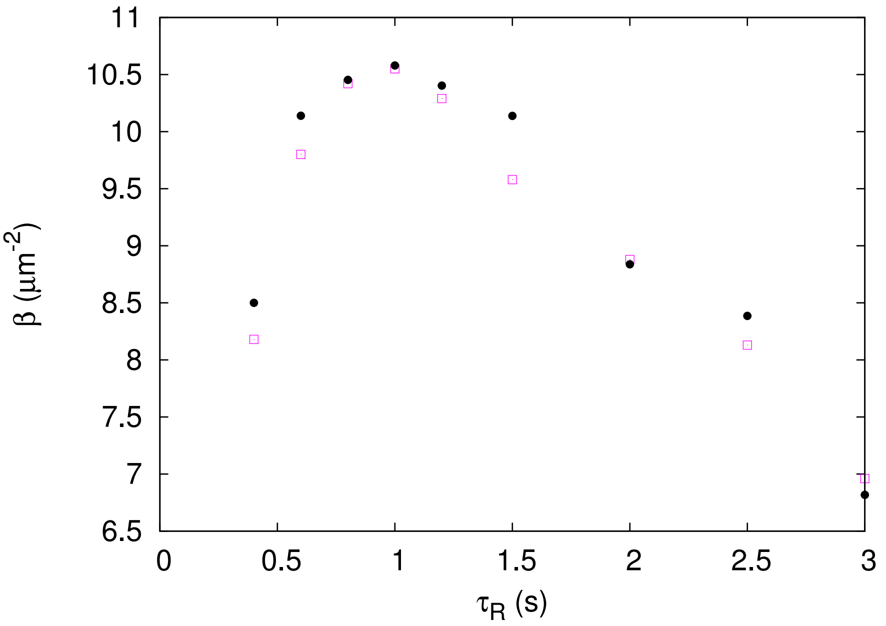

FIG. 6: The slope, β (scaled by a factor of 10^8), as a function of τ_R, in a non-linear model with the experimental bilobe response kernel of Fig. 1 in the paper. Here, $q = 0.5$ and the other numerical parameters are as in Fig. 3 in the paper. Comparison with Fig. 5 shows that the position of the maximum depends on the value of q. Open squares: numerical results from simulations. Solid circles: prediction of the coarse-grained model.

$1/\tau_T$, the master equations read

$$\begin{aligned} \frac{\partial R(x,t)}{\partial t} &= -v\partial_x R(x,t) + T^R(x,t)\tfrac{(1-q)}{\tau_T} \\ &\quad + T^L(x,t)\tfrac{q}{\tau_T} - R(x,t)\tfrac{1-\alpha c x}{\tau_0}, \end{aligned} \tag{2}$$

$$\begin{aligned} \frac{\partial L(x,t)}{\partial t} &= v\partial_x L(x,t) + T^L(x,t)\tfrac{(1-q)}{\tau_T} \\ &\quad + T^R(x,t)\tfrac{q}{\tau_T} - L(x,t)\tfrac{1-\alpha c x}{\tau_0}, \end{aligned} \tag{3}$$

$$\frac{\partial T^R(x,t)}{\partial t} = R(x,t)\tfrac{1-\alpha c x}{\tau_0} - T^R(x,t)\tfrac{1}{\tau_T}, \tag{4}$$

$$\frac{\partial T^L(x,t)}{\partial t} = L(x,t)\tfrac{1-\alpha c x}{\tau_0} - T^L(x,t)\tfrac{1}{\tau_T}. \tag{5}$$

We consider perfectly reflecting boundary conditions at $x = 0$ and $x = L$. This implies that, in the steady state, we must have $R(x) = L(x)$. The steady-state (total) density at location

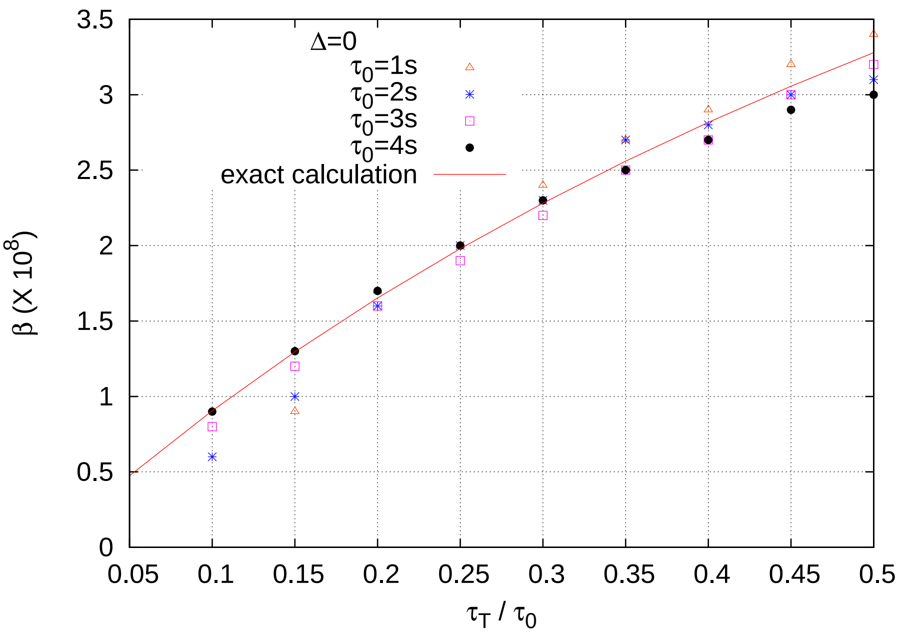

FIG. 7: *The steady-state slope β as a function of τ_T/τ_0 for $\Delta = 0$ and unmodulated tumbling. The solid line corresponds to the exact result from Eq. 7. Here we have used $L = 1000\mu m$, $\alpha = -0.1$, $q = 0.4$, $c = 0.001\mu m^{-1}$, $v = 10\mu m/sec$.*

x then becomes

$$N(x) = R(x) + L(x) + T^R(x) + T^L(x) = \frac{2}{L}\frac{1}{2+\frac{\tau_T}{\tau_0}(2-\alpha)}\left(1+\frac{\tau_T}{\tau_0}(1-\alpha c x)\right). \tag{6}$$

Therefore, the slope of the steady-state density profile is given by

$$\beta = G\left(\frac{\tau_T}{\tau_0}\right) = -2\alpha c \frac{1}{L}\frac{\tau_T}{\tau_0}\frac{1}{2+\frac{\tau_T}{\tau_0}(2-\alpha)}. \tag{7}$$

In Fig. 7, we compare this result with the slope measured in simulations. This plot demonstrates that, even in the absence of any memory or modulation of tumbling durations, it is possible to obtain chemotaxis in the steady state.

This result for the slope is slightly different for the case in which tumbling durations are modulated: then, τ_T in the above master equations is replaced by $\tau_T/(1+\alpha c x)$. Solving for the steady state, we find a total density

$$N(x) = R(x) + L(x) + T^R(x) + T^L(x) = \frac{1}{L\left(1+\frac{\tau_T}{\tau_0}\right)}\left(1+\frac{\tau_T}{\tau_0}\frac{1-\alpha c x}{1+\alpha c x}\right). \tag{8}$$

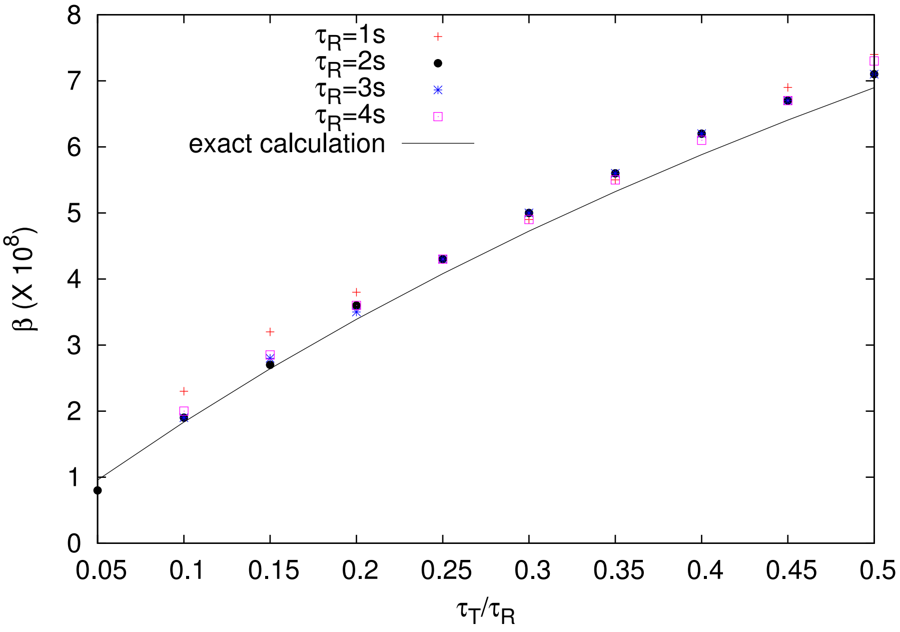

FIG. 8: *The slope, β, as a function of τ_T/τ_R for $\Delta = 0$ and modulated tumbling. Simulation parameters are $L = 1000\mu m$, $\alpha = -0.1$, $q = 0.4$, $c = 0.001\mu m^{-1}$, $v = 10\mu m/s$.*

For $\alpha c x \ll 1$, this is approximated by a linear form and the slope becomes

$$\beta = G\left(\frac{\tau_T}{\tau_0}\right) = -\frac{2\alpha c}{L}\frac{\tau_T}{\tau_0}\frac{1}{1+\frac{\tau_T}{\tau_0}(1-\alpha)}. \tag{9}$$

We compare this analytical result with simulations in Fig. 8, which shows a systematic deviation for large argument. We have verified that this mismatch originates in the linearizing approximation step from Eq. 8 to Eq. 9.

For $\Delta \neq 0$ and when both runs and tumbles are modulated, we measure the density profile in numerical simulations. For $R(t) = \alpha\delta(t-\Delta)$, our numerics indicate that the steady-state slope, β, is a sum of the Markovian component, G, defined in Eq. 9, and a non-Markovian component, F, which depends on Δ/τ_R but is independent of τ_T:

$$\beta = F\left(\frac{\Delta}{\tau_R}\right) + G\left(\frac{\tau_T}{\tau_R}\right). \tag{10}$$

In Fig. 9, we exhibit this scaling form as a function of τ_T/τ_R for a fixed, non-vanishing value of Δ/τ_R. Our results suggests that β is made up of two contributions: one from the modulating runs, encoded in F, and one from non-instantaneous tumbles, encoded in G. The latter contribution is independent of Δ.

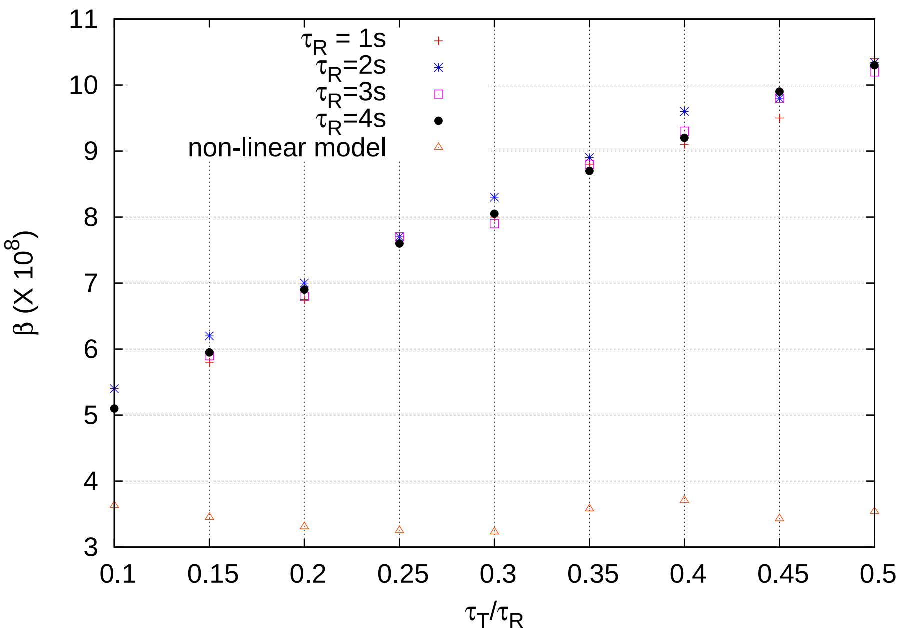

FIG. 9: *The scaling collapse of the slope, β, as a function of τ_T/τ_R for fixed value of Δ/τ_R. We have used $\Delta/\tau_R = 0.5$ here. The other simulation parameters are as in Fig. 8. We also plot the slope, β, in the non-linear model with $R(t) = \alpha\,[\delta(t-\Delta_1) - \delta(t-\Delta_2)]$, $\Delta_1 = 0s$ and $\Delta_2 = 1.5s$ and $\tau_R = 1s$: β does not show any significant dependence on τ_T.*

Finally, we can infer more general results from the simple form in Eq. 10. In particular, for an adaptive response function in the linear model, the positive and negative parts of the response function cancel out the effect of non-vanishing tumble durations. In this case, the steady-state slope, β, becomes independent of τ_T. Interestingly, we find the same applies even in the non-linear model (see Fig. 9).
